# Appropriate Use of Transesophageal Echocardiogram for Infective Endocarditis: A Single Center Experience

**DOI:** 10.1155/2019/7670146

**Published:** 2019-10-23

**Authors:** Robby Singh, Moumita Naidu, Yousef Bader, David Freeman, Marcel Zughaib

**Affiliations:** Providence Hospital, Michigan State University, Department of Cardiology, 16001 W Nine Mile Rd., Southfield, East Lansing, MI 48075, USA

## Abstract

**Background:**

Transesophageal echocardiogram (TEE) is a valuable tool in healthcare today with its ease of use, ability to visualize important structures not seen on transthoracic echocardiogram (TTE), and the relatively lower cost of TEE, high yield, and no significant radiation exposure. The American Society of Echocardiography (ASE) has developed an appropriate use criteria for use of TTE and TEE, which outline various scenarios where a TEE is indicated as an initial diagnostic testing modality and when it is useful as an adjunctive test in hopes of decreasing inappropriate use. Using these criteria as a guide, we devised a quality assessment study to investigate how well TEEs performed at our institution fit the appropriate use criteria specifically for the diagnostic workup of infective endocarditis.

**Methods:**

A retrospective chart review was performed for all TEEs performed in 2017 with the indication of endocarditis. Baseline patient characteristics, presence of bacteremia, and the quality of the TTE preceding the TEE were noted, as well as whether a vegetation, abscess, or perforation was visualized. We also determined if there was a cardiology consultation placed prior to TEE and if the patient had met the definition for endocarditis as defined by the Duke criteria. Finally, we made note of the TEE findings and assessed whether the TEE met appropriate use criteria developed by the American Society of Echocardiography.

**Results:**

A total of 50 patients who underwent TEE with the indication of “endocarditis” were identified. 36% of the TTEs prior to the TEE were rated as good quality, 40% as adequate, 4% as fair, 4% as suboptimal, 12% as technically difficult, and 4% were not rated. Vegetations were visualized on 12% of TTEs, 6% of patients had a prosthetic valve, and 6% had a cardiac device. In 20% of the cases, there was no cardiology consultation prior to the TEE and in 20% of the cases, there was no documented bacteremia. 26% of patients met the Duke criteria for endocarditis prior to TEE. Only 36% of TEEs revealed evidence of infection and of the patients with no evidence of infection, only 38% met appropriate use criteria. Overall, only 56% of patients met appropriate use criteria for TEE.

**Conclusion:**

Transesophageal echocardiography is a valuable tool in a modern physician's arsenal for managing a variety of diseases and conditions. However, the procedure is not without associated risks and its ease of use and widespread adoption has led to frequent questionable appropriateness of use of the test. Only 56% of the TEEs performed in our analysis met appropriate use. More awareness and education is needed for the appropriate use criteria for transesophageal echocardiography as outlined by the ASE to help reduce patient exposure to procedure related complications and to decrease medical costs on unnecessary procedures.

## 1. Introduction

Transesophageal echocardiogram (TEE) is a valuable tool in healthcare today with its widespread use ranging across a wide spectrum of clinical settings including critical care, other inpatient as well as outpatient. In addition to visualization of important structures not seen on transthoracic echocardiogram (TTE), a TEE can help assess aortic dissection, endocarditis, intracardiac thrombus, intracardiac shunting, and cardiac malignancies among other pathologies. The relatively lower cost of TEE compared to cardiac MRI and significantly reduced radiation exposure when compared to cardiac CT scan [1] have also contributed to large-scale use of TEE for diagnostic purposes.

While a TEE is a relatively safe procedure, it nonetheless remains an invasive procedure with potential associated complications. Upper GI complications associated with probe insertion include dental trauma, tonsillar bleeding, jaw subluxation, esophageal perforation, rupture of esophageal varices [2], and splenic laceration due to deep gastric insertion of the probe [3]. Aspiration may occur in obese patients, and probe insertion has also been known to promote sympathetic and parasympathetic reflexes leading to hypertension, hypotension, tachyarrhythmias, bradyarrhythmias, and myocardial infarction [4].

With these and many other procedure-associated complications in mind, the American Society of Echocardiography has developed an appropriate use criteria for use of TTE and TEE [5]. These expansive criteria outline various scenarios where a TEE is indicated as an initial diagnostic testing modality and when it is useful as an adjunctive test. Using these criteria as a guide, we devised a quality assessment study to investigate how well TEEs performed at our institution fit the appropriate use criteria specifically for the diagnostic workup of infective endocarditis.

## 2. Methods

A retrospective chart review was carried out for all TEEs performed in 2017 at our institution with the indication of endocarditis. During the data-gathering phase, patients' age, gender, race, and comorbidities were identified, including if the patient had a history of prosthetic valve, cardiac device, and endocarditis. The quality of the TTE preceding the TEE was noted, as well as whether a vegetation, abscess, or perforation was visualized. We also noted if the patient had documented bacteremia prior to the study and what organism was the cause of the bacteremia. We also determined if there was a cardiology consultation placed prior to TEE and if the patient had met the definition for endocarditis as defined by the Duke criteria. Finally, we made note of the TEE findings and assessed whether the TEE met appropriate use criteria developed by the American Society of Echocardiography.

## 3. Results

**Figure 1:**
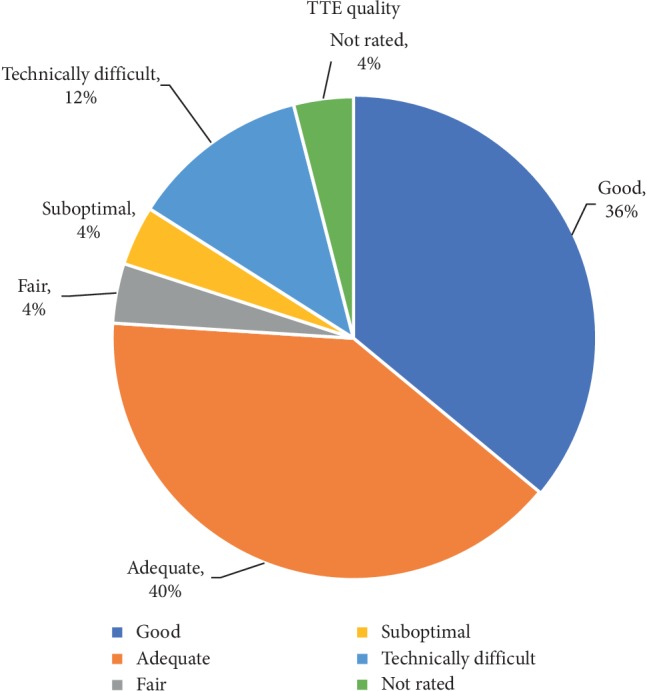
Quality of transthoracic echocardiogram prior to transesophageal echocardiogram.

A total of 50 patients who underwent TEE with the indication of “endocarditis” were identified and included in the analysis. Our institution is a tertiary care facility with both teaching faculty for residents and fellows as well as private physicians. The average age of the patients was 60 ± 17 years (Table 1). 72% (*n*=36) of the patients were male, 56% (*n*=28) were Caucasian, and 34% (*n*=17) were African-American. 36% (*n*=18) of the TTEs prior to the TEE were rated as good quality, 40% (*n*=20) as adequate, 4% (*n*=2) as fair, 4% (*n*=2) as suboptimal, 12% (*n*=6) as technically difficult, and 4% (*n*=2) were not rated (Figure 1). Vegetations were visualized on 12% (*n*=6) of TTEs, and only one study had a structure suspicious for but not definitive for a vegetation. 16% (*n*=8) of patients had a prosthetic valve and 6% (*n*=3) of patients had a cardiac device, with one patient overlapping between the 2 groups. Only 10% (*n*=5) of patients had a prior history of endocarditis.

**Figure 2:**
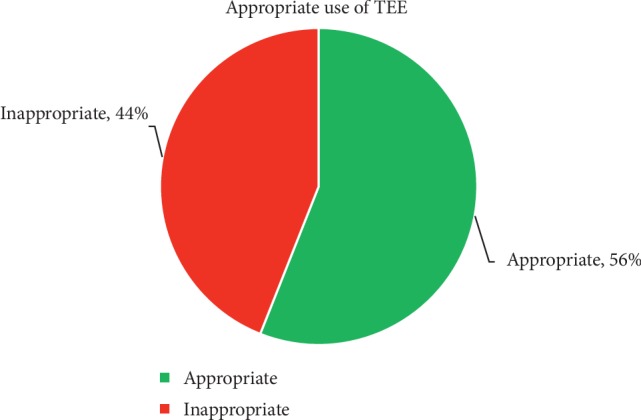
Appropriate use of TEE for the indication of infective endocarditis.

Interestingly, in 20% of the cases, there was no cardiology consultation prior to the TEE, and in 20% of the cases, there was no documented bacteremia (Table 2). The most common cause of infection was *Staphylococcus aureus* in 28% (*n*=14) of patients. 26% (*n*=13) of patients met the Duke criteria for endocarditis while an additional 4 patients met 1 major criteria and 1 or 2 minor criteria. Only 36% (*n*=18) of TEEs revealed evidence of infection and of the 32 patients with no evidence of infection, only 38% (*n*=12) met appropriate use criteria. Of those patients who did not have prior evidence of infection and did not meet TEE appropriate use criteria, only 2 patients, or 4%, were found to have evidence of vegetation on TEE. Overall, only 56% (*n*=28) of patients included in our analysis met appropriate use criteria for TEE (Figure 2).

## 4. Discussion

The value of TEE for evaluating cardiac function was first described in the early 1970s with the first M-mode echocardiogram reported in 1976 [5]. Rigid endoscopes needed for performing the procedure initially limited the widespread use of the technology though improvement in transducer designs, introduction of biplane and multiplane views, color and spectral Doppler, and overall improvements in transducer form factor have helped make the technology more commonplace with roughly 5–10% of patients in the cardiovascular ultrasound imaging lab undergoing TEE [5].

Not surprisingly, as a consequence of the technology becoming more commonplace, more procedures may be potentially ordered for uncertain indications. Though not as accurate as a TEE, a TTE has been noted to have a sensitivity of 61% and specificity of 94% for detecting vegetations and a negative likelihood ratio of 0.42, which improves when evaluating patients without prosthetic valves [6]. At our institution, which is a tertiary referral facility with academic faculty and residency and fellowship programs, we sought to determine the level of appropriateness of ordered TEEs. We chose to focus specifically on the workup of endocarditis as an indication for the purposes of our analysis.

Overall, only 56% of patients included in our analysis met appropriate use criteria for TEE. There are many potential explanations for this finding. A common indication for TEE is a TTE with poor-quality images, though in our analysis, 76% of the patients had TTE images that were rated as either good or adequate, but a TEE was still ordered and performed. 80% of the patients had documented bacteremia though only 26% of patients met the Duke criteria for endocarditis. As a conjecture, it is possible that a cause for these findings is the assumption that for a patient with bacteremia and a normal TTE, a TEE is still necessary to help rule out a diagnosis of infective endocarditis. Interestingly, in 20% of the patients, there was no documented bacteremia, yet a TEE was still performed. However, the actual diagnostic yield of such an approach is quite low, as only 1 patient had documented bacteremia, a normal TTE, and did not meet appropriate use criteria for TEE but still underwent TEE which found a vegetation. There was another instance where a patient did not have documented bacteremia but TTE showed possible aortic vegetation and the patient underwent a TEE not meeting appropriate use criteria and was found to have a vegetation on the aortic valve. Therefore, only 2 patients (4%) in our analysis who did not have evidence of bacteremia but still underwent TEE were found to have evidence of endocarditis. Though a TEE is a relatively safe procedure, it is not without associated adverse events, and also has the inherent risk associated with sedation needed for the procedure, which may be catastrophic in septic patients.

With the above findings in mind, we propose a quality improvement plan to help reduce the number of inappropriate TEEs for infective endocarditis. First, physicians and other providers must be given further education in the Duke criteria for endocarditis and made aware that a TEE is not necessary for diagnosing infective endocarditis and the diagnosis is less likely if there is no documented bacteremia. While primary care and infectious disease physicians may appreciate the benefits of TEE, awareness of the side effects and appropriate use of the test is limited many times to those performing the procedure. Accordingly, we recommend an earlier cardiology consultation. Not surprisingly, 20% of the patients in our study had no cardiology consultation prior to the procedure. Having a cardiologist involved early on in the care of the patients prior to ordering a TEE could potentially help decrease patients' exposure to procedure-related complications and unnecessary health care expenses. It is unclear why such a high number of patients did not have a cardiologist involved in their care but it reinforces the point that education about appropriate use for TEEs is not just important for cardiologists, but also primary care physicians. However, it must also be noted that in 80% (*n*=40) of the TEEs in our study, a cardiologist was consulted, and only 53% (*n*=21) of these patients fulfilled the appropriate use criteria indicating that more education and awareness of the appropriate use criteria is necessary amongst cardiologists as well.

## 5. Conclusion

Transesophageal echocardiography is a valuable tool in a modern physician's arsenal for managing a variety of diseases and conditions. However, the procedure is not without associated risks and its ease of use and widespread adoption has led to frequent questionable appropriateness of use of the test. Our analysis revealed that only 56% of TEEs performed at our institution with the indication of infective endocarditis met the appropriate use criteria as outlined by the American Society of Echocardiography. The inappropriate use of TEE unnecessarily increases the already large US healthcare expenditure and can expose patients to procedure-related complications that could otherwise be avoided. More awareness and education is needed for not only primary care and other referring physicians, but also cardiologists, of the appropriate use criteria for transesophageal echocardiography as outlined by the American Society of Echocardiography and the potential significant adverse events associated with the procedure.

## Figures and Tables

**Table 1:** Baseline characteristics of the patient population.

Age	60 years (±17 years)
Gender	56% male, 44% female
Ethnicity	56% Caucasian, 36%
Prosthetic valve	6%
Cardiac device	6%
History of endocarditis	10%

**Table 2:** Findings prior to transesophageal echocardiogram.

Vegetations visualized on TEE	12%
Cardiology consultation	80%
Documented bacteremia	80%
Met Duke criteria for endocarditis	26%

## Data Availability

The data used to support the findings of this study are available from the corresponding author upon request.
